# Effects of Plastic Waste on the Heat-Induced Spalling Performance and Mechanical Properties of High Strength Concrete

**DOI:** 10.3390/ma13153262

**Published:** 2020-07-23

**Authors:** Abrahão Bernardo Rohden, Jessica Regina Camilo, Rafaela Cristina Amaral, Estela Oliari Garcez, Mônica Regina Garcez

**Affiliations:** 1 Environmental Engineering Post-Graduation Program, Department of Civil Engineering, Regional University of Blumenau, Blumenau 89030-001, Brazil; arohden@furb.br (A.B.R.); eng.jessicaregina@gmail.com (J.R.C.); rafa.2796@hotmail.com (R.C.A.); 2 Faculty of Science Engineering and Built Environment, School of Engineering, Deakin University, Geelong 3216, Australia; estela.o@deakin.edu.au; 3 Civil Engineering Post-Graduation Program: Construction and Infrastructure, Interdisciplinary Department, Federal University of Rio Grande do Sul, Porto Alegre 90035-190, Brazil

**Keywords:** plastic waste, high strength concrete, spalling, residual mechanical properties

## Abstract

This paper investigates a potential application of hard-to-recycle plastic waste as polymeric addition in high strength concrete, with a focus on the potential to mitigate heat-induced concrete spalling and the consequent effects on the mechanical properties. The waste corresponds to soft and hard plastic, including household polymers vastly disposed of in landfills, although technically recyclable. Mechanical and physical properties, cracking, mass loss, and the occurrence of spalling were assessed in high strength concrete samples produced with either plastic waste or polypropylene fibers after 2-h exposure to 600 °C. The analysis was supported by Scanning Electron Microscopy and X-Ray Computed Tomography images. The plastic waste is composed of different polymers with a thermal degradation between 250 to 500 °C. Polypropylene (PP) fibers and plastic waste dispersed in concrete have proved to play an essential role in mitigating heat-induced concrete spalling, contributing to the release of internal pressure after the polymer melting. The different morphology of plastic waste and polypropylene fibers leads to distinct mechanisms of action. While the vapor pressure dissipation network originated by polypropylene fibers is related to the formation of continuous channels, the plastic waste seems to cause discontinuous reservoirs and fewer damages into the concrete matrix. The incorporation of plastic waste improved heat-induced concrete spalling performance. While 6 kg/m^3^ of plastic increased the mechanical performance after exposure to high temperature, the incorporation of 3 kg/m^3^ resulted in mechanical properties comparable to the reference concrete.

## 1. Introduction

The growing public concern on excessive plastic waste generation and poor final disposal results in global commitments to the production of recyclable, reusable, or compostable industrial products [1]. On the other hand, although technically recyclable, most types of plastic packaging are economically impossible to recycle and are expected to remain so for the foreseeable future [2], which induces product manufacturers to use new high-quality plastic bought at a lower cost [3]. As a result, in many countries, municipal recycling programs collect but do not recycle plastic waste, which results in tons of plastic being incinerated, sent to landfills, or even being made available to enter the oceans [4]. Another concern is that the disposal of plastic waste in landfills causes the pollution and contamination of soil and water, which subsequently can lead to human health-related problems due to the lead and cadmium content [2,5].

The annual production of plastics increased about 200-fold from 1950 (2 million tons) to 2015 (381,000 tons) and from the all plastics ever produced, only about 9% have been recycled, 12% incinerated, and 79% accumulated in landfills or the natural environment [6]. Mismanaged plastic waste rates of 56% to 90% were reported by Jambeck et al. [4] in countries where plastics represent 5% to 17% of the produced solid waste. Brazil, the largest country in Latin America, recycles only 13% of the 160 thousand tons of solid waste generated daily in the country [7], and the rate of discarded recyclable waste has reached 35% [8]. However, even with the enormous amounts of mismanaged recyclable plastic produced worldwide, there is a lack of investigations focused on the use of the discharged fraction of recyclable plastic waste; nevertheless, this strategy could effectively contribute to preventing environmental pollution [9].

The construction sector is one of the major contributors to CO_2_ emissions and waste generation [10,11] but shows excellent potential to incorporate waste, especially plastic, to produce new materials [12]. In this sense, although the discarded fraction of recyclable plastic waste is not commonly used as raw material to produce environmentally friendly construction materials, recent studies report potential benefits of plastic waste to improve mechanical properties of conventional [13,14,15], lightweight [16,17], self-compacting [5,18,19,20], and recycled aggregate [21,22,23] concrete. The most common applications are the use of plastic waste as a fine or coarse aggregate replacement [2,17].

Value-added applications of plastic waste in concrete buildings and paving blocks [24], fiber-reinforced concrete beams [25], and concrete footpaths [26], have also been investigated. The application of plastic waste in the production of High-Strength Concrete (HSC) has not been sufficiently studied, even though the use of HSC has been growing exponentially worldwide. One of the few studies on the use of plastic waste in structural concrete, conducted by Thorneycroft et al. [27], points out that the production of C50 with 10% of sand replacement by processed polymeric waste has the potential to save 820 million tons of sand every year. More recently, reused E-waste plastics have been successfully used in India to replace coarse aggregate in C40 and C60 HSC [28,29].

Regarding the use of HSC in reinforced concrete structures, the main concern is the increased tendency for heat-induced concrete spalling [30,31,32] when submitted to high temperatures, mainly due to its dense matrix and refined pore structure. Heat-induced concrete spalling is a thermo-hydro-mechanical coupled process related to thermal dilation and pore pressure gradients [33]. When submitted to high temperatures, the free water within the concrete turns into vapor, leading to vapor pressure bubbles and, consequently, tensile stresses that surpass the ones supported by the concrete. Yermack et al. [34] elucidate that as the water vapor moves both to the outside and to the colder parts of the concrete, fully saturated regions originate by condensed steam inside the concrete element, restricting the movement of water vapor and causing high tensile stresses that lead to concrete spalling. The heat-induced concrete spalling phenomenon is highly influenced by the moisture content, compactness, and porosity of the concrete matrix. The type of aggregate, shape and size of structural elements, high thermal gradients, non-uniform moisture distribution, and high reinforcement rate [25,26,27] also influence the heat-induced concrete spalling. Whilst the developing pore pressure is the main factor causing instantaneous damage to concrete exposed to high temperature, the concrete chemical decomposition causes additional damage when critical temperatures are reached [35,36]. Chemical and physical reactions that occur at elevated temperatures are also responsible for reductions in thermal conductivity and the specific heat of concrete [37].

Several technological solutions have been adopted to ensure safety the of reinforced concrete structures designed with HSC: limiting the use of HSC to low fire exposure risk structural elements, considering the use of external fire protection systems, or even reducing thermal stresses on the structural elements through design options [33]. On the other hand, synthetic (polypropylene—PP, water-soluble polyvinyl acetate—PVA) and natural (sisal, basalt) fibers dispersed into the concrete matrix have been successfully applied in concrete aiming at modifying its microstructure to mitigate the effects of heat-induced concrete spalling [33,35,38,39,40,41,42,43].

The main mechanisms behind fiber effectiveness to mitigate the heat-induced spalling are related to the formation of discontinuous reservoirs, continuous channels, and vacated channels in the concrete upon reaching the fiber melting point [32,35,44]. Such reservoirs and channels act as a vapor pressure dissipation network and allow water vapor and gases to be evacuated from the concrete structure [45]. As a result, pore pressure in concrete is released, and spalling is reduced [46]. Heat-induced spalling is a complex phenomenon due to the various mechanisms involved and the uncertainties around the potential mechanisms behind the effectiveness of the different materials used for spalling mitigation [32], which justifies further research. In addition to this, the use of plastic waste as dispersed fibers in concrete has not been sufficiently studied [2], and the use of plastic waste instead of synthetic fibers may contribute to reducing, at the same time, the environmental impacts derived from the production of industrialized fibers and the HSC cost per m^3^ [47].

Thus, this paper investigates a potential application of plastic waste as a polymeric addition in HSC, with a focus on the potential to mitigate heat-induced concrete spalling and the consequent effects on the mechanical properties. The plastic waste corresponds to the polymeric fraction of the discarded recyclable waste gathered in a waste collectors’ cooperative in Southern Brazil. Physical and mechanical properties, crack width, mass loss, and the evidence of heat-induced concrete spalling were monitored in concrete samples produced with plastic waste (PW) exposed to high temperatures. The results are compared to the ones of concrete samples produced with commercial polypropylene (PP) fibers. Scanning Electron Microscopy (SEM) and X-Ray Computed Tomography (CT) images supported the analysis of experimental data, endorsed by two-way ANOVA (analysis of variance) and Student t-test with α 0.05.

## 2. Experimental Program

### 2.1. Plastic Waste: Preparation, Identification, and Characterization

The discarded plastic waste used in this experimental program was collected in a waste collectors’ cooperative of a medium-sized municipality with 496.8 km² and 1.5 million residents in Southern Brazil.

The assessment of a representative sample of about 4 tons of solid waste sorted by the same collector’s cooperative over one year [48] quantified 30.5% (28.8 tons/month) of discarded recyclable waste. Apart from polymeric waste, a substantial presence of hazardous organic matter, construction and demolition waste, health care waste, electronics, textiles, footwear, batteries, and bulbs were identified. Three different classes of polymers were identified as plastic waste: (i) type 1, recyclable that could be commercialized by the cooperative, (ii) type 2, recyclable but not commercialized by the cooperative, and (iii) type 3, contaminated by solvents, paints, and other solids and liquids. Polymers identified as type 2 and 3 were used in this experimental program and represent about 14% of the recyclable waste sorted by the cooperative. The main components of type 2 and 3 waste are supermarket packaging, plastic film packaging, kitchen plastic film, soft drink bottle, plastic oil bottles, plastic bottles for sanitary products, ice cream plastic packaging, plastic cup, styrofoam, tubes, plastic packaging from snacks and cookies, plastic packaging from bakeries, packaging with paint or solvent, lunch boxes, composite plastic with metal, and composite plastic.

**Figure 1:**
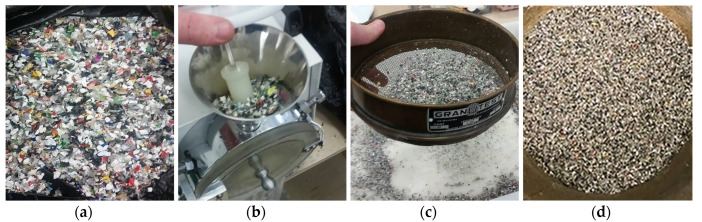
Plastic waste: (**a**) initial aspect; (**b**) milling; (**c**) sieving process; (**d**) final aspect.

Comminution processes were applied to reduce the plastic waste size (Figure 1). The polymers were first placed in a ∅ 300 × 305 mm rotating drum magnet separator (Metalmag, São Paulo, Brazil) to eliminate any metallic contamination. Then, two knife mills (Rone NFB 4315, São Paulo, Brazil, and Tecnal Willye TE-65, Piracicaba, Brazil) were used to produce plastic waste with similar size to commercial fibers used in concrete (passing through #2 mm sieve).

The identification of plastic waste constituents was carried out based on usual appearance, density, and behavior on heating including, transparency, consistency, flammability, the appearance of flame, and odor of vapors, as proposed by Braun [49].

Thermogravimetric analysis and Differential Scanning Calorimetry were performed to characterize the melting point (T_m_) and thermal degradation temperatures of the plastic waste using a Shimadzu DTG-60 equipment (Shimadzu, Tokyo, Japan) under Argon, following the ASTM 3850-19 and ASTM 3418-15 [50,51].

### 2.2. Production of High-Strength Concrete

Concrete mixes (Table 1) were designed to achieve a compressive strength of 60 MPa at 28 days, according to the mix proportioning method proposed by Aitcin [52]. The following materials were used: Brazilian cement CPV–ARI [53], similar to Portland cement Type III-HESC [54], Rice Husk Ash (RHA, specific gravity 2.16 g/cm^3^, loss on ignition 3%, 93% of SiO_2_), crushed gravel (gneiss, specific gravity 2.80 g/cm^3^, D_max_ 19 mm), sand (natural, specific gravity 2.60 g/cm^3^, fineness 2.81), polycarboxylate superplasticizer, and 2% entrapped air. Commercial PP fibers (PP, diameter 20 µm, length 10 mm, specific gravity 0.90 g/cm^3^, tensile strength 810 N/mm^2^, modulus of elasticity 2.890 N/mm^2^, melting temperature 160 °C, and ignition temperature 365 °C) and plastic waste (PW) were added to concrete mixes with dosages of 3 kg/m^3^ (0.125% by volume) and 6 kg/m^3^ (0.250% by volume).

Concrete mixes were produced in a planetary mixer with materials added in the following order: cement, rice husk ash, sand, 50% of water, gravel, 50% of water, superplasticizer, PP or PW. The mixing process lasted approximately 5 min. Forty cylindrical ∅ 100 × 200 mm specimens were cast [55] for each concrete mix. Samples were kept for 24 h at room temperature, demolded, and transferred to a lime-saturated water tank for curing until the age of 28 days [56].

### 2.3. Heating–Cooling Procedure

**Figure 2:**
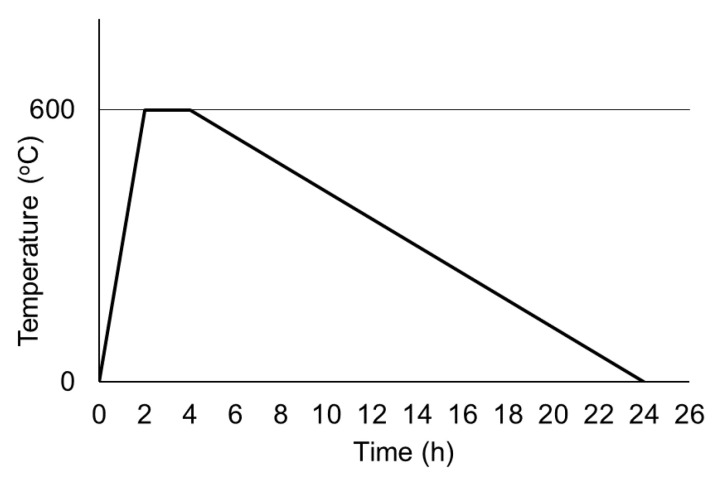
Temperature–time curve.

An electric muffle furnace was used to heat 20 saturated specimens of each mixture [36]. Heating–cooling cycles consisted of three phases: temperature rise at 5 °C/min [30,57,58,59,60], temperature dwell corresponding to two hours at 600 °C to achieve thermal steady-state [61] and slow cooling to room temperature at about 0.5 °C/min to avoid thermal shock (Figure 2). Saturated specimens were used to minimize possible effects of the moisture content on the heat and mass transfer properties of concrete, especially in the initial state [33].

### 2.4. Assessment of Concrete Behavior at High Temperature

**Figure 3:**
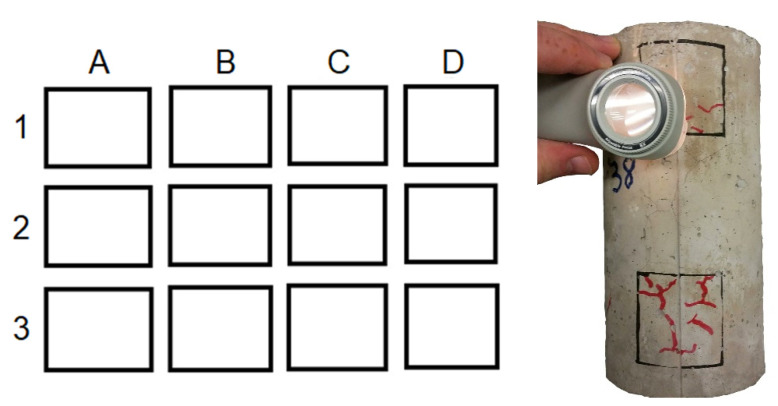
Cracking visual inspection pattern.

Heat-induced concrete spalling was assessed by cracking visual inspection and mass loss of specimens submitted to 600 °C for two hours. Cracking visual inspection was carried out in 10 randomly selected specimens of each concrete mix. The number of cracks with openings between 0.2 mm and 0.5 mm was quantified in 3 chosen demarcated areas of each specimen (upper, intermediate, and lower), according to the pattern presented in Figure 3. Heat-induced concrete spalling severity was classified according to Table 2. Mass loss was determined by the difference in the masses of concrete specimens submitted to the high temperature before heating and after the slow cooling stage.

Water absorption by capillarity test [62], Scanning Electron Microscopy (SEM JEOL JSM-6060, Tokyo, Japan), and X-Ray Computed Tomography (micro-focus CT 150kV Hamamatsu X-ray source with a tungsten target and a flat panel detector C7942 120 × 120 mm^2^, 2240 × 2368 pixel^2^, pixel size 50 µm, Hamamatsu, Japan) images were performed to support the assessment of concrete behavior at high temperature. Porosity was quantified in reconstructed volumes using the volume analysis tool of Octopus V8.6 software (TESCAN, Brno, Czech Republic), based on a back-projection algorithm with convolution and correction for cone-beam. Young’s modulus [63], compressive strength [64], and tensile strength [65] tests were performed before and after exposure to 600 °C to identify possible changes in the mechanical properties of concrete specimens.

Table 3 shows a summary of the investigation tests. Statistically significant results were determined by two-way ANOVA and Student t-test with α 0.05.

## 3. Results and Discussion

### 3.1. Identification and Characterization of Plastic Waste

The identification process revealed that the plastic waste sample is composed by Polycarbonate (PC), High-Density Polyethylene (HDPE), Low-Density Polyethylene (LDPE), Polyvinyl Chloride (PVC), Polypropylene (PP), Acrylonitrile Butadiene Styrene (ABS), Polystyrene (EPS), and a low concentration of other plastics, according to Table 4. The sources of recycled plastic are shown in Table 4. The high amount of HDPE is a consequence of its low potential for recycling, while LDPE and PP are widely found in discarded recyclable waste due to the frequent use in food packs, plastic bags, and garbage bags.

The thermal degradation of plastic waste samples extends to near 500 °C with a high mass loss (Table 5). These results endorse the mechanism described by Maluk et al. [32], in which the volumetric and phase changes of polymers during heating may lead to microcracks into the concrete matrix and the creation of discontinuous reservoirs that enhance water vapor migration within the concrete.

### 3.2. Heat-Induced Concrete Spalling

**Figure 4:**
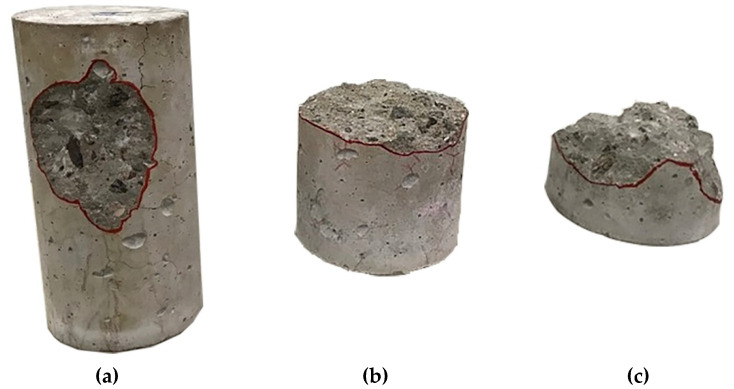
Spalling in CREF specimens: (**a**) Level 2–Medium severity; (**b**,**c**) Level 3–High Severity.

**Figure 5:**
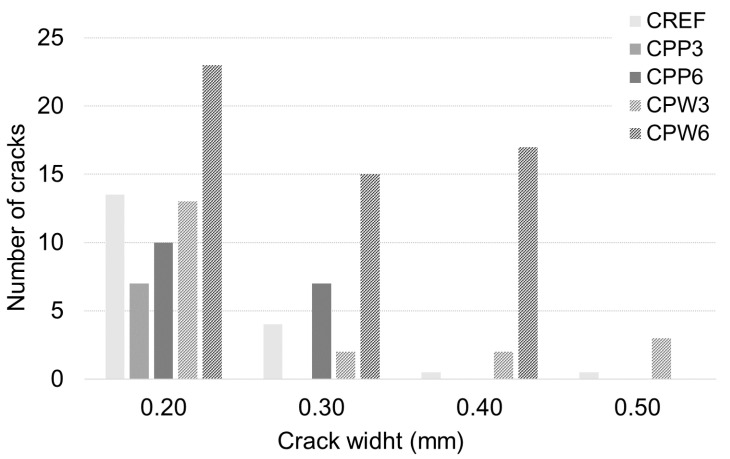
Cracking after exposure to high temperature.

**Figure 6:**
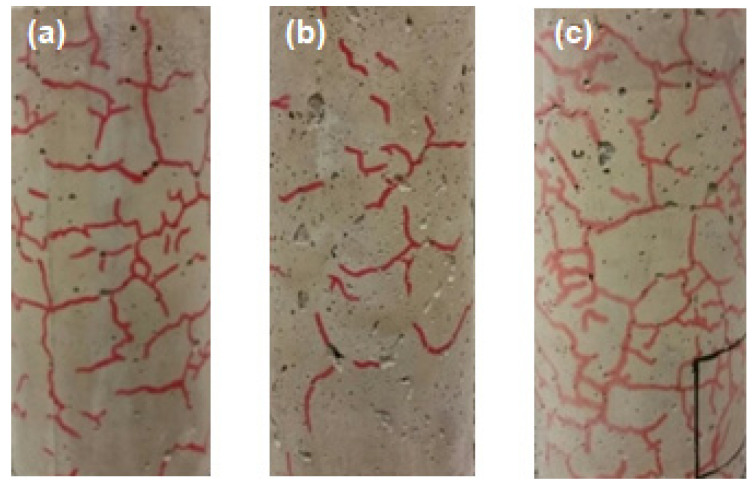
Distribution of cracks in the frontal view of concrete specimens: (**a**) CREF; (**b**) CPP3; (**c**) CPW3.

Table 6 presents a summary of the damages in concrete samples after the heating process. The spalling phenomenon was only observed in three reference concrete (CREF) specimens, with medium to high severity damages, as seen in Figure 4. Figure 5 shows the quantification of cracking in samples due to heating. The presence of PP fibers limited the cracking level at the surface of the concrete specimens. CPW3 and CREF specimens present a similar crack pattern (Figure 6). However, none of the CPW3 samples presented spalling. On the other hand, the CPW6 specimens presented the higher number of cracks of all samples. The higher the amount of PP fibers or PW, the higher the number of cracks observed on the surface of the concrete specimens. The results evidence the positive effects of both PP fibers and PW in mitigating heat-induced spalling phenomenon. All CPW and CREF samples presented higher crack width (0.1 to 0.5 mm) when compared to CPP (0.1 to 0.3 mm). Mass loss ratios (Table 6) also evidence the positive effects of the addition of 6 kg/m^3^ of plastic waste, which is statistically comparable to 3 kg/m^3^ and 6 kg/m^3^ of PP fibers, with a confidence level of 95%. Considering that the volume change is negligible after exposure to elevated temperatures [37], the mass loss may also be used as an indicator of changes in concrete density.

**Figure 7:**
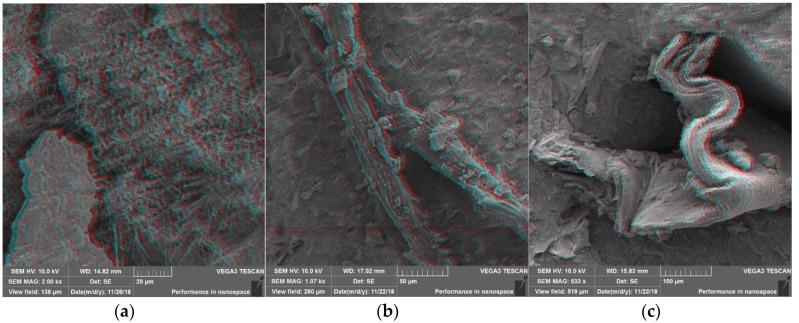
Scanning electron microscopy (SEM) 3D anaglyph images: (**a**) Reference concrete matrix; (**b**) PP fiber in concrete matrix; (**c**) plastic waste (PW) in concrete matrix (SEM images without anaglyph effect presented in Figure S1 of the supplementary material).

**Figure 8:**
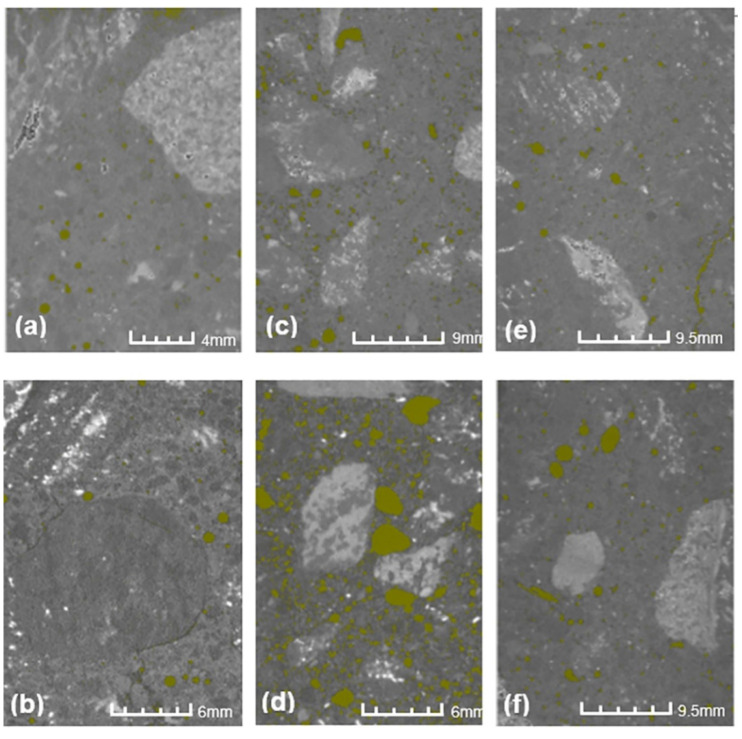
Air voids identification in computed tomography (CT) images: (**a**) CREF before heating; (**b**) CREF after heating; (**c**) CPP3 before heating; (**d**) CPP3 after heating; (**e**) CPW3 before heating; (**f**) CPW3 after heating.

The SEM 3D anaglyph images in Figure 7 (better visualized with anaglyph glasses—red and cyan lens) illustrate the different morphology of PP fibers and PW, which helps to explain their distinct mechanisms of action in mitigating the heat-induced concrete spalling. CT images in Figure 8 suggest that while the vapor pressure dissipation network caused by PP fibers seems to be mainly related to the formation of continuous channels in the concrete matrix, PW seem to cause the discontinuous reservoirs mentioned by Maluk et al. [32] and Khoury and Willoughby [35].

**Figure 9:**
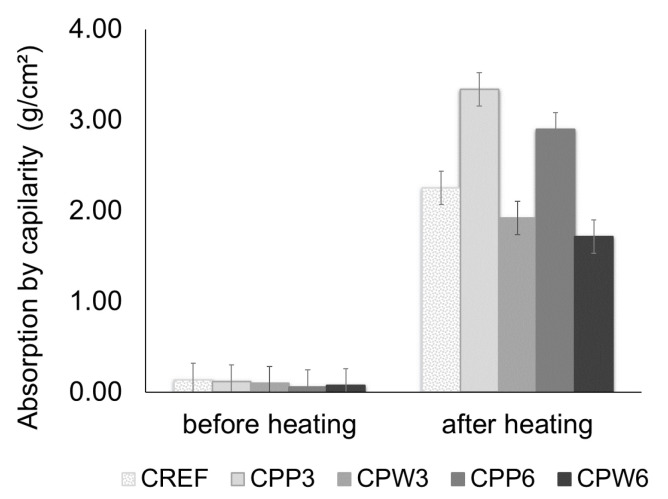
Absorption by capillarity before and after heating.

A rough estimate of porosity performed based on CT images (Figure 8) of concrete before and after heating shows no relevant changes for CREF (1.61% and 1.30%) and CPW3 (2.48% and 2.45%). Such results corroborate the similar behavior of CREF and CPW3 concerning the mass loss, cracking level on the concrete surface, and absorption by capillarity, as shown in Table 7 and Figure 9. However, it is important to highlight once again that CPW3 did not present heat-induced concrete spalling. The porosity of CPP3 increased from 3.02% to 8.95% after heating, which is consistent with its spalling performance: lower mass loss, lower cracking level on the concrete surface, and higher absorption by capillarity (Table 7, Figure 9) when compared to CREF and CPW3.

The results of absorption by capillarity bring some insights on the porosity and permeability characteristics of concrete samples before and after the heating process, which is important since the heat-induced concrete spalling phenomenon is extremely related to the capillary pore structure [58]. The absorption by capillarity of concrete samples, after heating, increased 16-fold for CREF, 28 for CPP3, 48 for CPP6, 18 for CPW3, and 22 for CPW6. The results obtained with absorption by capillarity before and after heating are consistent with the occurrence of cracks at the paste–aggregate interface as a result of hydrate decomposition and thermal mismatch between the different polymers, aggregates, and matrix at a high temperature [34,66,67], besides the thermodynamic conditions reached in the porous network of each concrete mixture.

**Figure 10:**
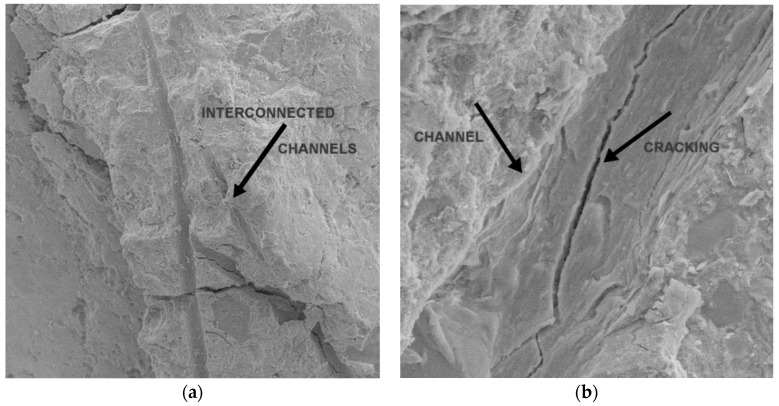
Formation of channels and cracking in the concrete matrix of CPP3 sample: (**a**) 400X; (**b**) 3000X.

The results show no statistically significant difference for absorption by capillarity of samples before heating. After heating, the absorption by capillarity of concrete samples with PP fibers is statistically higher than CREF, which is related to the presence of larger and interconnected pores and channels [34], as shown in Figure 10.

CPW samples showed lower absorption by capillarity when compared to CREF after heating, which could be related to the surface morphology of the plastic waste and potentially less internal damage caused by the exposure to high temperature [1,68]. Ozawa and Marimoto [39] reported that the morphology of the polymeric addition might help to prevent the development of high vapor pressures within the concrete. The melting and vaporization of PP fibers could lead to stress concentration at the tip of the fibers, resulting in additional cracking into the concrete matrix [34,40]. The lower absorption by capillarity provides evidence that this concentration might be significantly reduced with the incorporation of plastic waste instead of PP fibers. It is interesting to note that, even with lower absorption by capillarity, the performance of CPW6 is better than the reference concrete and comparable to that of CPP3 and CPP6 fibers in terms of non-occurrence of spalling and mass loss.

### 3.3. Mechanical Properties

Table 8 illustrates the contribution of PP fibers and plastic waste on the strength and stiffness of concrete samples and the influence on the residual properties after the exposition to high temperature. Except for concrete mixture CPP3, all of the results of compressive strengths are higher than the target f_ck_ of 60 MPa (CREF 73.47 MPa, CPP6 72.54 MPa, CPW3 63.19 MPa, CPW6 66.74 MPa).

As addressed in Table 1, superplasticizer was used to satisfy a slump range of 200 mm ± 20 mm, keeping the water/cement ratio constant. The visual inspection of concrete fragments extracted from the specimens after the mechanical tests did not detect significant agglomeration regardless of the percentage of PP fibers and plastic waste (0.125% or 0.250% by volume).

Even though the compressive strength of CPW3 is 10.20% lower than CREF at ambient temperature, after exposure to a high temperature, both compressive strengths are statistically equivalent, evidencing the positive effects of the plastic waste addition. For CPW6, the contribution of plastic waste is even more evident, since the compressive strength is 7.8% lower than CREF at ambient temperature and 49.51% higher after exposition to high temperature. Additionally, the compressive strength of CPW6 and CPP6 exposed to high temperatures are statistically equivalent.

While 3 kg/m^3^ of PP fibers and plastic waste did not show any significant difference to CREF, the incorporation of 6 kg/m^3^ increased the tensile strength by 100.98% (CPP6) and 29.41% (CPW6) compared to CREF. The most prominent result is the tensile strength of CPW6, 49.51% higher than CREF after exposure to high temperature, while other concrete mixtures do not show any significant difference when compared to CREF.

Lower Young’s modulus resulted in higher residual modulus, which corroborates to the findings of Xiong and Liew [69] and Ali et al. [70]. Fire tests in prestressed concrete beams performed by Selvaggio and Carlson [71] revealed that a combination of a high modulus of elasticity with reduced tensile and compressive strengths of concrete contribute to the lack of resistance to bursting stresses that leads to concrete spalling.

These results demonstrate the potential of plastic waste, especially considering the mechanical properties after heating, which corroborates to the findings of mitigation of heat-induced spalling phenomenon presented in Section 3.2.

**Figure 11:**
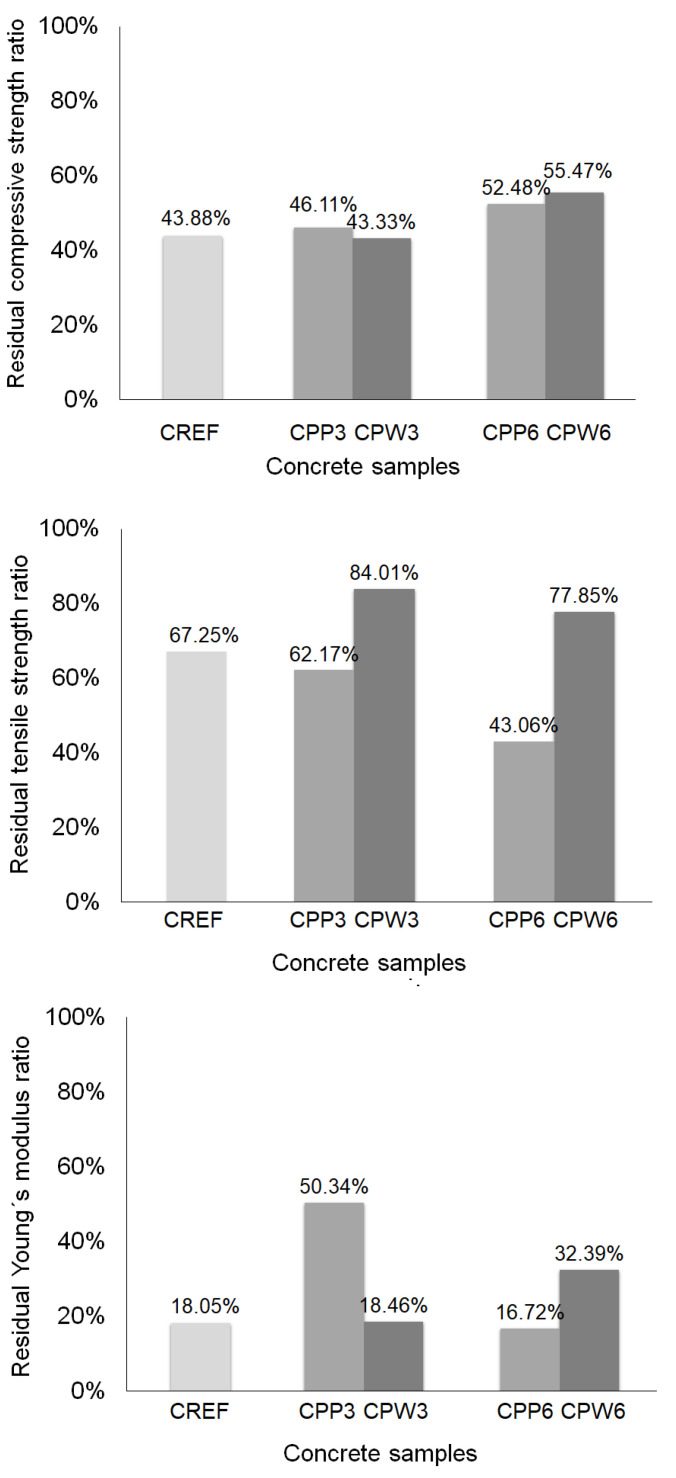
Residual mechanical properties.

Figure 11 shows the quantitative relationship between the residual mechanical properties of the concrete mixtures with different contents of PP fibers and plastic waste, considering the respective properties at ambient temperature. The assessment of residual mechanical properties provides important information for structural safety evaluation and definition of repair methods for fire-damaged reinforced concrete structures [72].

A post-fire compressive strength factor of 0.45 is recommended in the general rules for the structural fire design of Eurocode 2 [73] for normal-weight concrete with siliceous aggregates exposed to 600 °C. The reduction in compressive strength occurs due to the degradation of C-S-H after 400 °C [74] and the decomposition of Portlandite between 200 °C and 600 °C [75,76,77], which is considered the limit for the integrity of concrete mechanical properties. The results of Figure 11 demonstrate that only the concretes with the incorporation of PP fibers and plastic waste were able to reach the 0.45 factor, with the higher residual compressive strengths obtained with the incorporation of 6 kg/m^3^ of polymeric addition. Georgali and Tsakiridis [77] reported that the compressive strength typically ranges from 55% to 70% of its original value; however, the maximum exposure temperature was 550 °C.

As PP fibers and plastic waste melt at temperatures of about 200 °C, a reduction in the tensile strength of CPP and CPW samples to the same level of CREF would be expected after exposure to 600 °C temperatures [62]. The results of Figure 11, however, show that the residual tensile strength of CPW is higher than CPP and CREF. Kodur [40] and Neville [75] point out that the residual tensile strength of concrete is crucial since cracking in real structures generally occurs due to tensile stresses, and the structural damage of the member in tension is often generated by progression in microcracking. The tensile-strength behavior of CPW samples after exposure to high temperature demonstrate the positive effects achieved with the incorporation of plastic waste in high-strength concrete.

The results of Figure 11 show that losses in Young’s modulus are about 80%, while the losses in compressive and tensile strengths are about 60% and 30%, respectively, for reference concrete. The incorporation of polymers seems to change these relationships, which demands more investigation for further consideration in design codes related to concrete structures. In the literature, Young´s modulus losses are related to the evaporation of capillary and adsorbed water, dehydration of C-S-H, and removal of non-evaporable water [78]. The results of Figure 11 show that concrete samples with lower Young’s modulus resulted in higher residual Young’s modulus after heating.

The overall results of residual mechanical properties demonstrate the similar performance of CPW3 and CREF, although the tensile strength of CPW3 is not as severely affected by high temperature as it is for CREF. It is noteworthy that CPW6 showed the lowest losses in mechanical properties after exposure to high temperature.

## 4. Conclusions

This paper investigated the effects of plastic waste on the heat-induced spalling performance and mechanical properties of high strength concrete. The main findings are summarized below.

- Plastic waste dispersed in concrete helped mitigate heat-induced concrete spalling by releasing internal pressure after polymer melting.
- The vapor pressure dissipation network caused by PP fibers are mainly related to the formation of continuous channels, while plastic waste caused discontinuous reservoirs that lead to less damage in the concrete matrix.
- The residual mechanical performance of concrete with 3 kg/m^3^ of plastic waste is comparable to the reference concrete, with the advantage of heat-induced concrete spalling mitigation.
- The incorporation of 6 kg/m^3^ not only improved the heat-induced concrete spalling, but also the mechanical performance after exposure to high temperature.

These results are limited to this case study since the heat-induced concrete spalling is highly dependent on experimental and mix design parameters. Workability, dispersion, and durability issues related to the use of plastic waste in HSC should be further investigated to confirm the trends reported in this paper.

## Figures and Tables

**Table 1:** Concrete mix proportion.

Concrete *	Cement kg/m^3^	RHA kg/m^3^	Sand kg/m^3^	Crushed Gravel kg/m^3^	Water L/m^3^	Admixture ** L/m^3^	PP kg/m^3^	PW kg/m^3^
CREF	450	50	708	1000	143	up to 11	-	-
CPP3							3	-
CPP6							6	-
CPW3							-	3
CPW6							-	6

* target f_ck_ = 60 MPa; ** enough superplasticizer to satisfy a slump range of 200 mm ± 20 mm; CREF: reference concrete; CPP3 and CPP6: concrete with 3 kg/m^3^ and 6 kg/m^3^ of PP fibers; CPW3 and CPW6: concrete with 3 and 6 kg/m^3^ of plastic waste.

**Table 2:** Spalling classification.

Spalling Classification	Identification of Spalling Severity *
Level 1 Low Severity	Low popping sound. Detachment of small concrete fragments off the specimen surface with consequent formation of small grooves.
Level 2 Medium Severity	Medium popping sound. Detachment of concrete fragments off the specimen surface with consequent. formation large fragmented areas and damaged edges.
Level 3 High Severity	Sudden loud noise. Explosive rupture accompanied by failure of the concrete specimen.

* adapted from Kirchhof et al., 2011, based on Malhotra, 1984 and Ali et al., 2004.

**Table 3:** Summary of investigation tests.

Investigation	Number of Specimens per Concrete Mixture	Heating Condition (Temperature °C)	Specimen Geometry (mm)
Cracking Visual Inspection	10	600	Cylindrical ϕ 100 × 200 mm
Mass Loss	20		
Water Absorption by Capillarity	20	Ambient-600	
Compressive Strength	13		
Tensile Strength	3		
Young’s Modulus	4		

**Table 4:** Plastic waste composition.

Polymers	Concentration (%)
PC	26.14
HDPE	21.12
LDPE	13.25
PVC	13.09
PP	11.35
ABS	11.21
EPS	2.64
Other Plastics	1.20

**Table 5:** Thermal characterization of plastic waste.

Group	Source	T_m_* (°C)	Thermal Degradation		
			Beginning (°C)	End (°C)	Mass Loss (%)
PP	Metalized Plastic	130.22	362.30	462.17	93.27
PVC, LDPE, HDPE, ABS	Hard Plastic, Pipes	175.30	362.83	433.54	99.27
		174.03	397.45	457.90	93.12
PP, HDPE	Plastic Bag	174.07	333.27	415.76	96.85
		176.90	354.56	410.02	93.30
PP	Wire	167.41	359.64	460.78	99.52
		174.76	403.06	462.39	98.83
HDPE	Black Garbage Bag	130.88	430.49	483.07	95.45
	Ration Bag	130.50	430.92	486.59	94.44
PVC	Plastic Pool	167.13	258.62	331.54	73.78
PC, PVC	Clear Transparent Plastic	261.96	413.15	461.95	99.88
		260.37	404.84	471.76	99.79

**Table 6:** Summary of the damages in concrete specimens.

Concrete	Heat-Induced Spalling *			Cracking Level on the Concrete Surface **	Mass Loss (%) ***
	Low	Medium	High		
CREF	-	✔	✔	+++	6.43 ^a^
CPP3	-	-	-	+	5.91 ^b^
CPP6	-	-	-	++	5.43 ^c^
CPW3	-	-	-	+++	6.51 ^a^
CPW6	-	-	-	+++++	5.79 ^b,c^

* According to the classification presented in Table 2; ** According to the number of cracks between 0.2 and 0.5 mm accounted in the demarcated areas of the cracking visual inspection scheme presented in Figure 3; *** Excluding the 2 highly damaged specimens of CREF showed in Figure 5b,c; + to ++++ indicates low to high cracking level; Same letters in the same column represent equivalent means.

**Table 7:** Absorption by capillarity.

Concrete	Absorption by Capillarity * (g/cm^2^)	
	Before Heating	After Heating
CREF	0.14 ^a^	2.26 ^a^
CPP3	0.12 ^a^	3.37 ^c^
CPP6	0.06 ^a^	2.89 ^d^
CPW3	0.10 ^a^	1.89 ^a,b^
CPW6	0.07 ^a^	1.58 ^b^

* at 72 h; In each column, the same letters represent equivalent means.

**Table 8:** Mechanical properties.

Concrete	Compressive Strength *fc_j_* (MPa)	Residual Compressive Strength *frc_j_* (MPa)	Tensile Strength *ft_j_* (MPa)	Residual Tensile Strength *frt_j_* (MPa)	Young’s Modulus *E* (MPa)	Residual Young’s Modulus *E_r_* (MPa)
CREF	85.94 ^a^	37.71 ^a^	3.06 ^a^	2.06 ^a^	47.09 ^a,d^	8.50 ^a^
CPP3	61.05 ^d^	28.15 ^c^	3.52 ^a^	2.19 ^a^	37.38 ^c^	19.12 ^c^
CPP6	92.29 ^b^	48.43 ^d^	6.15 ^b^	2.65 ^a^	50.72 ^d^	8.48 ^a^
CPW3	77.17 ^c^	33.44 ^a^	3.01 ^a^	2.53 ^a^	45.57 ^a^	8.41 ^a^
CPW6	79.21 ^c^	43.94 ^b,d^	3.96 ^c^	3.08 ^b^	41.77 ^b^	13.53 ^b^

Average results; In each column, the same letters represent equivalent means.

## Supplementary Materials

The following are available online at https://www.mdpi.com/1996-1944/13/15/3262/s1, Figure S1: SEM images of Figure 7 without anaglyph effect: (a) Reference concrete matrix; (b) PP fiber in concrete matrix; (c) PW in concrete matrix.
