# Supplementary material for: Effects of Plastic Waste on the Heat-Induced Spalling Performance and Mechanical Properties of High Strength Concrete
Source: Materials (Basel). 2020 Jul 23;13(15):3262. doi: 10.3390/ma13153262 (PMC7435736; doi:10.3390/ma13153262)
Supplement: Supplementary file 1 [file materials-13-03262-s001.pdf]

materials

Supplementary Material

Effects of Plastic Waste on the Heat-Induced Spalling Performance and Mechanical Properties of High Strength Concrete

Abrahão Bernardo Rohden [1], Jessica Regina Camilo [1], Rafaela Cristina Amaral [1], Estela Oliari Garcez [2] and Mônica Regina Garcez [3,*]

1. Environmental Engineering Post-Graduation Program, Department of Civil Engineering, Regional University of Blumenau, Blumenau 89030-001, Brazil; arohden@furb.br (A.B.R.), eng.jessicaregina@gmail.com (J.R.C.), rafa.2796@hotmail.com (R.C.A.)
2. Engineering and Built Environment, Faculty of Science, Deakin University, Geelong 3216, Australia; estela.o@deakin.edu.au
3. Civil Engineering Post-Graduation Program: Construction and Infrastructure, Interdisciplinary Department, Federal University of Rio Grande do Sul, Porto Alegre 90035-190, Brazil

* Correspondence: monica.garcez@ufrgs.br; Tel.: +55-51-3308-1330

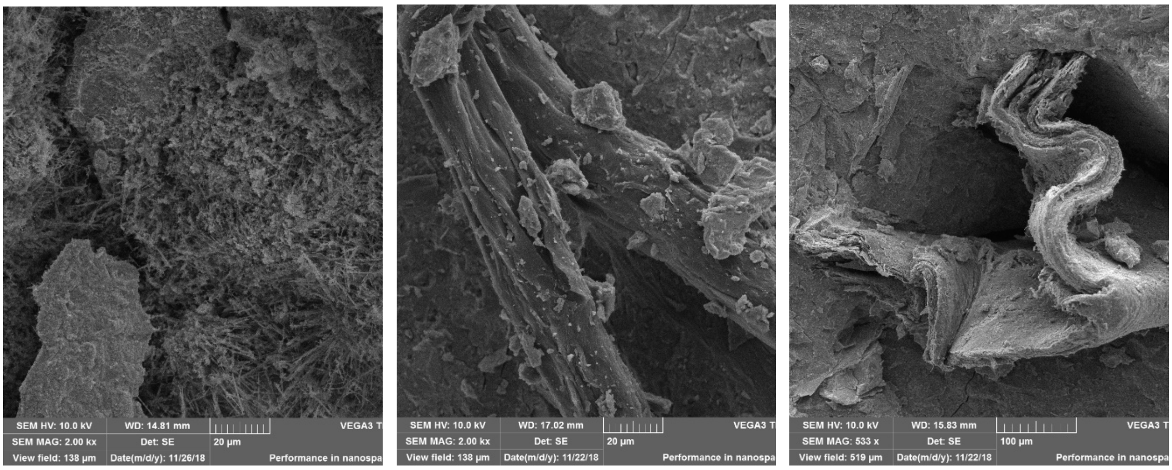

(a) (b) (c)

Figure S1. SEM images of Figure 7 without anaglyph effect: (**a**) Reference concrete matrix; (**b**) PP fiber in concrete matrix; (**c**) PW in concrete matrix.

© 2020 by the authors. Submitted for possible open access publication under the terms and conditions of the Creative Commons Attribution (CC BY) license (http://creativecommons.org/licenses/by/4.0/).
